# Immune Checkpoint Inhibitor-Induced Guillain Barre Syndrome: A Single-Institution Case Report and Narrative Review

**DOI:** 10.7759/cureus.61489

**Published:** 2024-06-01

**Authors:** Precious O Idogun, Said Hafez-Khayyata, Daniel Ezekwudo

**Affiliations:** 1 Hematology and Medical Oncology, Corewell Health William Beaumont University Hospital, Royal Oak, USA; 2 Pathology, Corewell Health William Beaumont University Hospital, Royal Oak, USA; 3 Hematology and Oncology, Corewell Health William Beaumont University Hospital, Royal Oak, USA

**Keywords:** autoimmune like, systemic literature review, immunotherapy, immune-checkpoint inhibitor adverse effects, nivolumab-related adverse events, nivolumab, immune-checkpoint inhibitors, guillain barre’s syndrome (gbs)

## Abstract

Guillain-Barré syndrome (GBS) resulting from the use of immune checkpoint inhibitors (ICIs) is relatively uncommon but has been reported. Herein, we discuss a case of a 67-year-old patient who received neoadjuvant ICI for treatment of non-small cell lung cancer and then presented with lower extremity weakness and areflexia, progressing to respiratory muscle and upper extremity weakness. Given the increasing use of ICI in cancer management, awareness of neurological autoimmune side effects is essential. ICI-mediated GBS can be severe and fatal if not diagnosed promptly. We discuss a case of ICI-induced GBS and review literature on current management approaches.

## Introduction

Over the past several years, immunotherapy has changed the landscape of the available treatments for several oncologic malignancies. Immune checkpoints can be manipulated through monoclonal antibody (MoAb) blockage of the checkpoint ligands, receptors, or both [1]. In 2011, ipilimumab, a human monoclonal cytotoxic T-lymphocyte-associated protein 4 (CTLA-4) antibody, was the first immune checkpoint inhibitor (ICI) approved for use in patients with metastatic or unresectable melanoma [2]. The success of ipilimumab in melanoma encouraged the development of other ICIs. Nivolumab is a fully human MoAb that binds to the programmed death-1 (PD-1) receptor with high specificity and affinity [3]. PDL1 is upregulated in various tumor types, including melanoma, non-small cell lung cancer (NSCLC), and squamous cell head and neck carcinomas, and is a major mechanism of immune evasion. MoAbs against both PD-1 and PD-L1 show clinical activity in various tumors [4]. PD-1 inhibitors, such as nivolumab, can activate T cells to kill tumor cells by blocking the binding of the PD-1 receptor and programmed death ligand 1 and ligand 2 (PD-L1 and PD-L2) [3]. Despite having significantly less toxicity compared to traditional chemotherapy, ICIs are not without adverse side effects. Collectively, these side effects are known as immune-related adverse events (irAEs). Patients experiencing irAEs usually have a broad spectrum of symptoms. It is important for clinicians to have a high level of suspicion for these irAEs to allow for prompt recognition and management.

Guillain-Barré syndrome (GBS) is an autoimmune-mediated disease in which most patients have a prodromal infection. Common infectious pathogens include cytomegalovirus, Epstein-Barr virus, influenza virus, human immunodeficiency virus, mycoplasma, *Haemophilus,* and *Campylobacter jejuni* [5]. Following the eradication of poliovirus, GBS is the most common cause of acute or subacute, flaccid neuromuscular weakness worldwide [6]. GBS patients often present with a severe and sudden onset course of symptoms that usually includes ascending weakness and non-length-dependent sensory symptoms [6]. Symmetric involvement is a key feature of GBS [6]. The acute progression of limb weakness, often with sensory and cranial nerve involvement one to two weeks after immune stimulation, proceeds to its peak clinical deficit in two to four weeks [7]. GBS is a clinically diagnosed disorder, but nerve conduction studies (NCS) can help to support the diagnosis and discriminate between axonal and demyelinating subtypes [7].

In general, patients with GBS need careful monitoring and supportive care. Up to 25-30% of patients may eventually require artificial ventilation; hence, admission and monitoring in an intensive care setting are essential [8].

## Case presentation

A 67-year-old female was diagnosed with stage IIB (cT1b, cN1, cM0) adenocarcinoma of the left lung. She was started on neoadjuvant pemetrexed, carboplatin with nivolumab [9]. She received three infusions of nivolumab, the last done 10 weeks before symptom onset. She underwent a left lower lobectomy two weeks prior to symptom onset.

Subsequently, the patient developed numbness in all four extremities. This was followed by gradually worsening weakness of her lower extremities. She also reported tremors and stated that the numbness/tingling was intermittently painful. Her symptoms progressed to the point where she was having difficulty walking and needed full assistance with standing or climbing stairs. Prior to presentation, she did not require any durable medical equipment for ambulation. 

Initial workup at an outside facility included computed tomography (CT) of the head, CT of the cervical spine, and computed tomography angiography (CTA) of the chest. These studies showed no acute abnormalities. Complete blood count (CBC) and comprehensive metabolic panel (CMP) were normal at that time as well. 

Symptoms persisted, so the patient presented two days later to our facility for further work-up and evaluation. She denied any recent unusual foods, camping, tick bites, sick contacts, diarrhea, or vomiting. She also denied dyspnea, dysphagia, or changes in her vision. 

Other medical history included ascending colon malignancy treated with colonic resection two years prior, lumbar spondylolisthesis with L4-5 posterior fusion and L4 laminectomy, osteoporosis, GERD, and emphysema. She had a 40-pack-year history of smoking but quit a few years prior to presentation. There was no history of alcohol or drug use. The patient was evaluated by a multidisciplinary team consisting of neurology, physical medicine/rehabilitation, and hematology/oncology. Physical exam on the initial presentation demonstrated stable vital signs with a blood pressure of 168/82, pulse rate of 86 beats per minute, oral temperature of 98.2°F (36.8°C), respiratory rate of 20 breaths per minute, and oxygen saturation of 99% on room air. The mental status and cranial nerve examination were all intact. She had no cerebellar symptoms. However, she demonstrated stocking-glove distribution of pin, light touch, temperature, proprioception, and vibration deficits in her bilateral upper extremities and bilateral lower extremities. She demonstrated 3/5 upper extremity strength and 2/5 lower extremity strength. She had total loss of reflexes in the biceps, brachioradialis, patellar, and achilles, with no plantar response.

**Figure 1:**
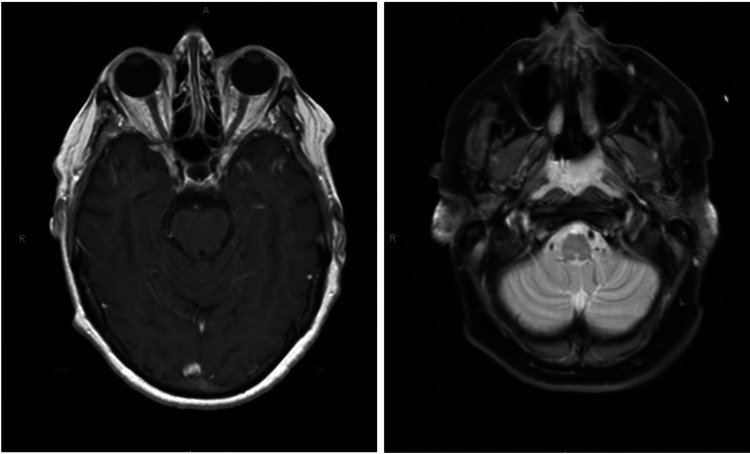
Brain MRI demonstrating chronic ischemic changes. MRI: magnetic resonance imaging.

The brain magnetic resonance imaging (MRI) (Figure 1) showed no evidence of an acute infarct. However, there were mild T2 and FLAIR signal hyperintensities in the periventricular deep white matter and subcortical white matter of the left frontal lobe and in the right hippocampus consistent with chronic ischemic change. There was no mass effect or midline shift and no evidence of pathological enhancement. Electromyography (EMG) was performed and showed severe axonal demyelinating sensorimotor polyneuropathy, which further confirmed suspicion for GBS or GBS variant (Table 1). MRI of the cervical, lumbar, and thoracic spine with and without gadolinium demonstrated postsurgical changes and degenerative changes with no high-grade central canal stenosis in the thoracic or lumbar region.

**Table 1:** EMG studies. EMG: electromyography; NL: normal; UTA: unable to activate; PSWs: positive sharp waves; Fibs: fibrillation potentials; Fasic: fasiculations; DI: dorsal interosseus muscle; ECR: extensor carpi radialis longus; VMO: vastus medialis oblique; TA: transversus abdominis; EHL: extensor hallucis longus; FDL: flexor digitorum longus; EDB: extensor digitorum brevis. *Decreased insertional activity.

Muscle	Innervation	Nerve roots	PSWs	Fibs	Fasic	Activation	Recruitment	Amplitude	Duration	Phases
Left										
Deltoid	Axillary	C5-6	3+	3+	0	NL	Decreased	NL	Increased	Increased
Biceps	Musculocutaneous	C5-6	0	0	0	NL	Decreased	NL	Increased	Increased
Triceps	Radial	C7	0	0	0	NL	Decreased	Increased	Increased	Increased
First, DI	Ulnar	C8-T1	0	0	0	NL	Decreased	NL	Increased	Increased
ECR	Radial	C6-7	0	0	0	NL	Decreased	NL	Increased	Increased
VMO	Femoral	L2-4	0*	0	0	Decreased	Decreased	NL	Increased	NL
TA	O peroneal	L4-5	2+*	2+	0	Decreased	Decreased (1 unit)	NL	Increased	NL
EHL	O peroneal	L5-S1	0*	0	0	Decreased	Decreased (1 unit)	Increased	Increased	Increased
FDL	Tibial	L5-S1	0*	0	0	UTA	---	---	---	---
Gastrocnemius	Tibial	S1-2	0*	0	0	NL	Decreased (1 unit)	NL	Increased	Increased
EDB	Deep peroneal	L4-5	0*	0	0	UTA	---	----	---	---

**Figure 2:**
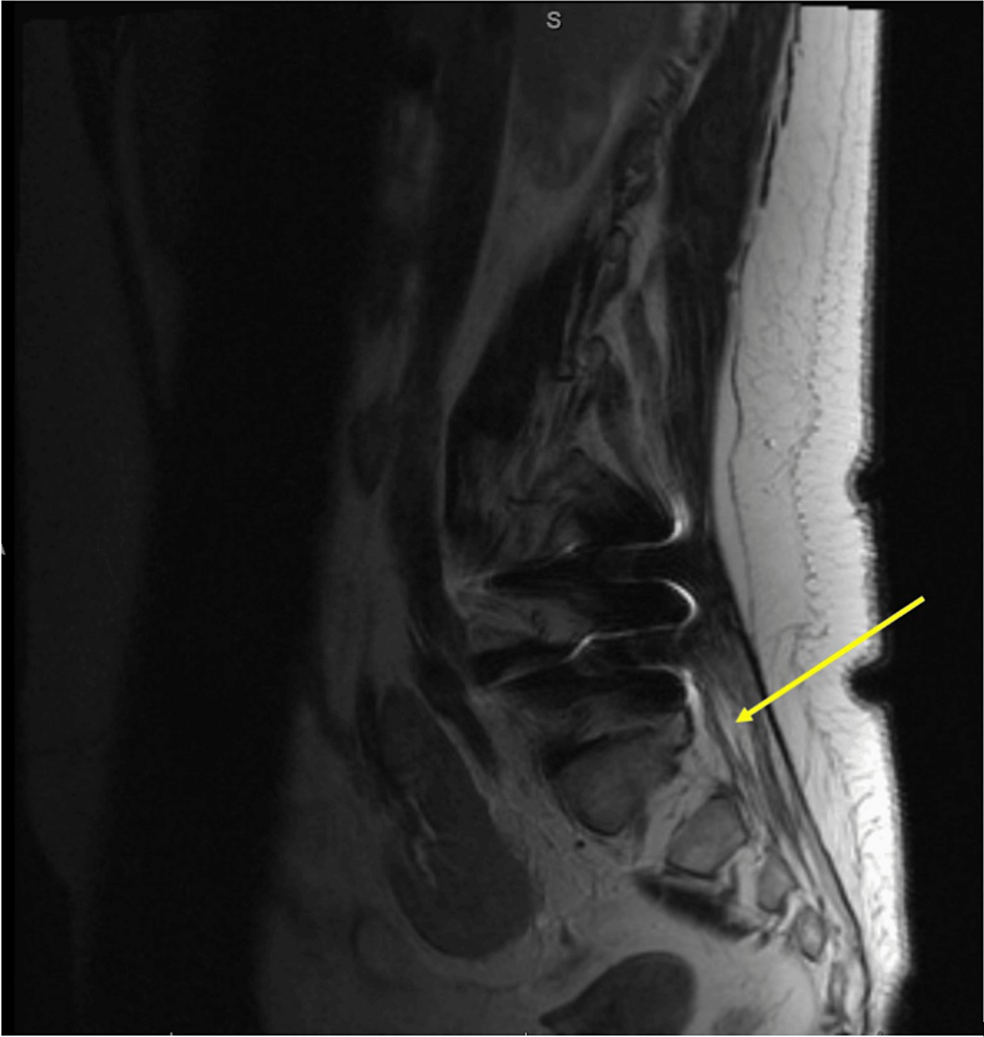
Lumbar MRI with and without gadolinium demonstrates postsurgical and degenerative changes as well as diffuse enhancement of all visualized cauda equina nerve roots. MRI: magnetic resonance imaging.

MRI of the cervical, lumbar, and thoracic spine with and without gadolinium demonstrated postsurgical changes and degenerative changes with no high-grade central canal stenosis in the thoracic or lumbar region. There was also diffuse enhancement of all visualized cauda equina nerve roots (Figure 2). Findings were thought to be likely related to a GBS/acute inflammatory demyelinating polyneuropathy type syndrome.

A lumbar puncture (LP) was performed, and it demonstrated albuminocytologic dissociation (Table 2), which was concerning for GBS. The motor and sensory neuropathy panels were performed as well, and this was negative. Flow cytometry showed absent B cells with a small number of T cells present, which had CD4:CD8 ratio of 1.1:1 with normal expression of CD5. Cerebrospinal fluid (CSF) culture showed no growth after 5 days. Gram stain showed rare PMNs with no organisms seen. Full CSF results are demonstrated in Table 2.

**Table 2:** Demonstration of LP results with CSF analysis showing characteristic albuminocytologic dissociation. CSF: cerebrospinal fluid; LP: lumbar puncture; PMNs: polymorphonuclear neutrophils.

CSF test	Analysis results	Reference range and units
Glucose	82	50-80 mg/dL
Protein	197	15-45 mg/dL
RBC	1	≤0/mcL
Color	Colorless	Colorless
Clarity	Clear	Clear
Total nucleated cells	3	≤5/mcL
Total number of cells seen on CSF scan	100	Variable
PMNs	10	Variable
Mononuclear cells	86	Variable
RBCs	4	Variable
CSF oligoclonal bands evaluation	Negative	Negative
IgG index	0.76	0.28-0.66 ratio
IgG	19.3	0.0-6.0 mg/dL
Albumin	131	0-35 mg/dL
IgG/albumin	0.15	0.09-0.25 ratio
Synthesis rate	39.0	≤8.0 mg/d
Albumin index	35.9	0.0-9.0 ratio
IgG, S	707	768-1632 mg/dL
Albumin, S	3654	3500-5200

On the second day of hospitalization, the patient reported worsening symptoms and dyspnea. Given the progression of symptoms, she was started on intravenous immunoglobulin (IVIG) and negative inspiratory force (NIF) was checked twice daily. The patient shortly demonstrated increased breathing with increasing oxygen requirements. Her NIF was noted to be low (-17 cm H_2_O). In addition, the patient reported a globus sensation. 

She was subsequently transferred to the Intensive Care Unit (ICU) and intubated for airway management due to concern for an impending airway collapse. The patient remained intubated for one week. In addition to her respiratory status, her neurologic status and weakness continued to worsen despite IVIG treatment. She then started on intravenous steroids while in the ICU. Eventually, she was able to transfer back to the floor after two weeks in the ICU. Initially, the patient was receiving methylprednisolone (40 mg BID), which was switched to the equivalent dose of dexamethasone while in the ICU. She eventually transitioned to oral prednisone 30 mg twice a day. She remained on this dose for 10 days with further tapering down to 20 mg BID for two weeks, then 15 mg BID for another two weeks. The patient gradually recovered neurologic function and was transferred to rehabilitation three weeks later, with eventual discharge seven weeks later. A three-month follow-up revealed that the patient had recovered all neurologic function with no complications during the recovery phase.

## Discussion

ICIs have provided newer options of therapy and have helped to achieve success in prolonging survival in different cancers. As of the time of drafting this paper, six ICIs (nivolumab, ipilimumab, pembrolizumab, cemiplimab, atezolizumab, and durvalumab) have been approved by the US Food and Drug Administration (FDA) for patients with NSCLC [10]. Clinical trials of ICIs in patients with NSCLC have shown superior overall survival (OS), median progression-free survival, and objective response rate (ORR) [11]. Despite being considered relatively less toxic compared to traditional chemotherapy, ICIs are not without their own side effect profiles due to their unique mechanism of action. Disinhibition of T-cell function by ICIs can lead to a variety of inflammatory side effects or irAEs [12]. 

Multiple mechanisms have been proposed to account for the development of irAEs, although the exact pathophysiology is not fully understood [12]. The dermatologic, gastrointestinal, pulmonary, hepatic, and endocrine systems are most frequently involved in irAEs [13]. In general, neurologic adverse events associated with ICIs are less common, and their reported prevalence varies widely. One study reported an incidence of <4% following treatment with anti-CTLA-4 antibodies, 6% with anti-PD-1 antibodies, and 12% with combination therapy involving both [13]. An analysis using a pharmacovigilance database reported an overall incidence as low as 0.93% of serious (>grade 3) neurologic irAEs (NirAEs) in patients with melanoma who were treated with nivolumab with or without ipilimumab [14]. NirAEs are an emerging area of interest because of the complexity of the nervous system and the potential for long-term morbidity [15]. Neuromuscular junction disorders and myositis are the most common NirAEs and account for approximately 70% of cases [16]. Based on a retrospective study in a tertiary care center, a vast majority of patients (89%) first developed a NirAE within 12 weeks (about three months) of initial therapy [17].

In a systematic review paper from China published in 2021, a total of 30 cases that reported GBS associated with ICI use were analyzed. Their analysis revealed that more than half (16/30) of these cases were associated with Nivolumab use, 11/30 were associated with ipilimumab, and 7/30 had received treatment with pembrolizumab [18]. GBS with potentially life-threatening consequences occurred in 0.1-0.2% of patients treated with ICIs [18]. In addition, they found a median time to death of 64.5 days among patients who died [18]. 

GBS is a group of autoimmune disorders manifested by acute polyradiculoneuropathy, and it is the most common cause of acute flaccid paralysis [6]. ICI-associated GBS is seen in 0.1%-0.3% of all patients receiving ICIs [19]. It has mostly been observed with the combination therapy of ipilimumab and nivolumab [20]. In a systemic review from 2021, GBS-like NirAE and other peripheral neuropathies (22%) were the second most common NirAEs after myositis [21]. 

ICI-related GBS should be suspected in patients on ICI treatment who develop subacute progressive weakness of the limbs, sensory loss, and areflexia. In general, GBS triggered by ICI is generally similar to GBS not associated with ICI in terms of presentation and clinical course [22] . The diagnosis of GBS is based on clinical history and examination and is supported by ancillary investigations such as CSF examination and electrodiagnostic studies [23]. 

However, in the presence of high clinical suspicion, it is recommended to begin treatment before the tests results. CSF examination may be used to rule out other causes of weakness. The classic CSF finding in GBS is the combination of an elevated CSF protein level and a normal CSF cell count (known as albuminocytologic dissociation) [24]. Normal CSF protein levels, however, do not rule out a diagnosis of GBS. There is little diagnostic value in measuring serum levels of anti-ganglioside antibodies as it is limited and assay-dependent [25]. A positive test result may be helpful if the diagnosis is in doubt, but a negative test result does not rule out GBS [25]. This indicates that the CSF protein analysis is a specific diagnostic tool but is not sensitive enough to be used alone. In addition, electrodiagnostic studies are not required to diagnose GBS but could be useful in supporting the diagnosis. MRI is also not part of the routine diagnostic evaluation of GBS. The presence of nerve root enhancement on gadolinium-enhanced MRI is a nonspecific but sensitive feature of GBS [26]. 

From our review of the literature, it appears that, at best, there is a modest response to initial IVIG therapy. However, subsequent treatment with high-dose corticosteroids is what drives the most improvement in motor weakness. According to the American Society of Clinical Oncology (ASCO) guidelines on ICI-associated toxicity, corticosteroids and IVIG are traditional treatments for ICI-associated GBS. The combination of these treatments can improve the clinical symptoms of GBS patients by 73% [27]. This is different from idiopathic GBS, in which corticosteroids do not provide any additional benefit [28]. Plasma exchange (PE) can be used as a second-line treatment if previous treatments are ineffective. However, the efficacy of plasmapheresis as a first-line treatment is unknown [29]. One case report in the literature reports improvement in ICIs-induced GBS with the use of mycophenolate mofetil [29].

A critical issue in clinical practice is the safety of resuming ICI therapy following the resolution of an adverse event. Prospective data from clinical trials are limited since most study protocols recommend discontinuation of ICI therapy if a serious adverse event occurs. A recent retrospective study involving patients with melanoma found that toxicity may be treatment-specific rather than generalizable across the several types of immune checkpoint blockade [30]. 

However, another retrospective study published in the Journal of Clinical Oncology found that in patients who developed irAEs and then improved, re-treatment with anti-PD(L)-1 therapy was associated with recurrence or new irAEs in half of the patients [31]. Overall, life-threatening toxicity, particularly cardiac, pulmonary, or neurologic toxicity, is considered a permanent contraindication to immunotherapy [32]. Given the substantial risk of permanent neurological damage in the case of NirAEs, we do not recommend attempting retreatment with an ICI. 

Physical therapy (PT) is a critical component of GBS rehabilitation and management in general. One paper reported a 20-week (about four and a half months) phase of intensive PT for a patient with GBS that eventually allowed for greater flexibility of static and dynamic postures, increased motivation, and the ability to walk without mobility assistance [33, 34]. In our case, the patient also required extensive PT spanning six weeks to regain complete neurological function. However, there are not enough high-quality data or randomized controlled trials to draw absolute conclusions about the effects of PT.

**Figure 3:**
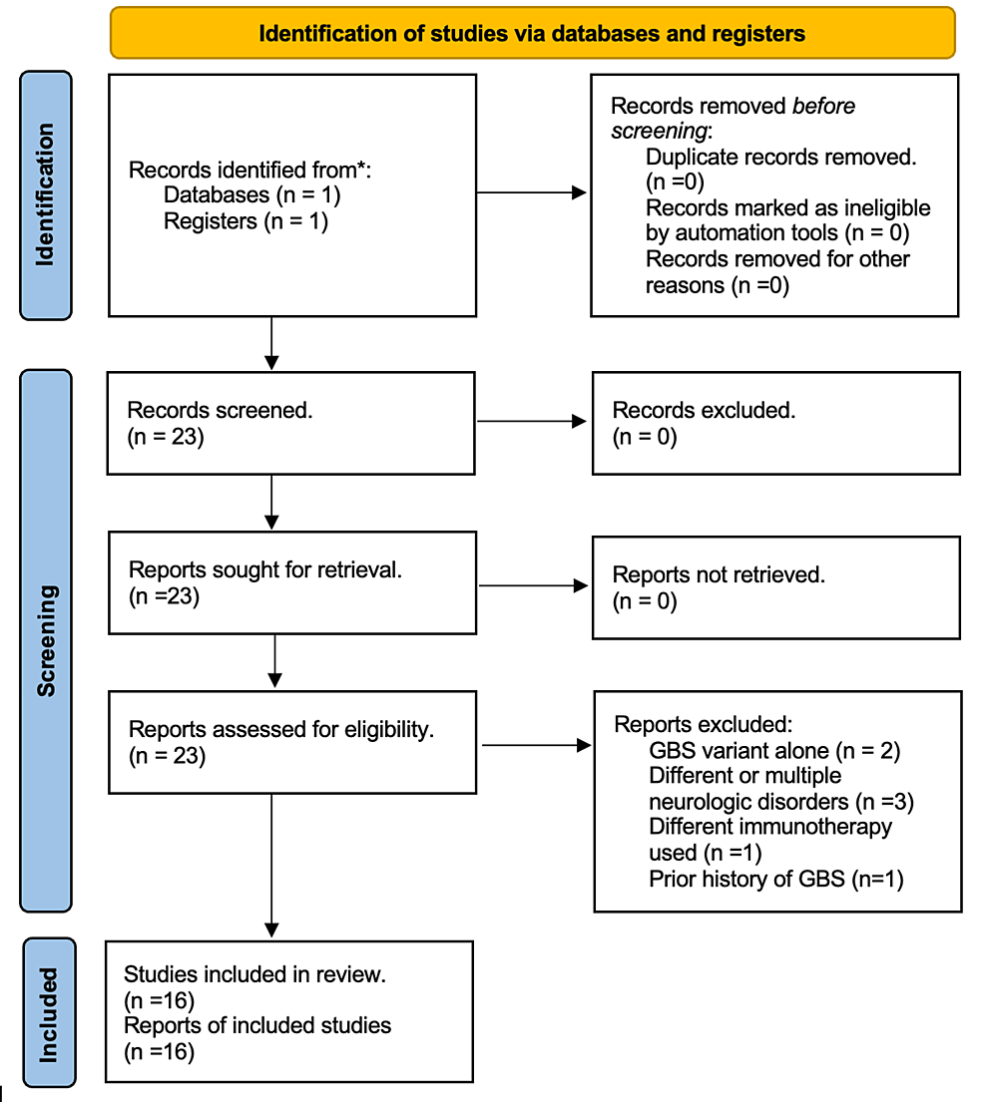
PRISMA 2020 flow diagram for a systematic review that includes searches of one database only. PRISMA: Preferred Reporting Items for Systematic reviews and Meta-Analyses.

A systematic literature search using the PRISMA guidelines was performed in PubMed for case reports of GBS associated with nivolumab. The date range of the articles included was between 2016 and 2023 and was limited to English language case reports. The keywords used were as follows: ((((((Guillain barre syndrome)) OR (acute inflammatory demyelinating polyradiculoneuropathy)) OR (Miller Fisher Syndrome)) OR (acute motor axonal neuropathy)) OR (acute motor-sensory axonal neuropathy)) AND (Nivolumab). Patients who received ipilimumab in addition to Nivolumab were not excluded. Figure 3 shows the PRISMA flow diagram. For each case, we extracted data on demographics and clinical manifestations. There were no duplicates identified. The final number of cases considered eligible was 16. An overview of all 17 cases (including our own) is presented in Table 3. 

**Table 3:** Main characteristics and diagnostic details of included cases. GBS: Guillain-Barré syndrome; ICI: immune checkpoint inhibitor; NSCLC: non-small cell lung cancer; RCC: renal cell carcinoma.

Article	Tumor type and stage	Age and sex	Time of GBS onset after ICI initiation	Therapy received for GBS	Patient outcome
Janssen et al., 2021 [27]	Malignant melanoma, stage IV	67; M	3 weeks	Prednisolone 2 mg/kg/d for 14 days and IVIG 0.4 g/kg/d for five days	Slow recovery of motor and sensory functions
Kyriazoglou et al., 2019 [34]	Invasive bladder cancer, stage IV	74; M	8 weeks	IVIG for five days along with prednisolone	Progressive improvement of neurological symptoms; residual areflexia; subsequent death
Tanaka et al., 2016 [35]	Malignant melanoma; stage IV	85; F	7.5 weeks	IVIG at 400 mg/kg/day for five days; prednisolone 1 mg/kg per body weight with gradual tapering	Slow complete recovery of motor and sensory function
Nukui et al., 2018 [36]	Nasal cancer; stage IV	45; M	10 weeks	IVIG; subsequent steroid pulse therapy	Complete recovery of neurologic function
Pierrard et al., 2019 [37]	Urothelial carcinoma; stage IV	70; M	58 weeks	IVIG 0.4 mg/kg once a day for five days and methylprednisolone 1 mg/kg for seven days	Complete recovery of neurologic function
Yuen et al., 2019 [38]	Malignant melanoma; stage IV	66; M	2 weeks	IVIG and prednisolone	Death
Jacob et al., 2016 [39]	NSCLC; stage IV	68; F	12 weeks	IVIG and plasma exchange	Death
Thapa et al., 2018 [40]	NSCLC; stage IV	60; M	2 weeks	Tapering dose of prednisolone was initiated; IVIGs were initiated when no response was seen	Worsening neurologic function with eventual respiratory compromise
Fukumoto et al., 2017 [41]	NSCLC; stage IV	66; M	5 weeks	Prednisolone 60 mg/day and IVIG 0.4g/kg for five days	Symptoms worsened Gradually improved three months later, he was able to walk with a cane
Mazzaschi et al., 2020 [42]	NSCLC; Stage IV	80; F	5 weeks	Course of IVIG at 400 mg/kg per day for five days; two weeks of 1 mg/kg prednisolone	Complete recovery of neurologic function
Schneiderbauer et al., 2017 [43]	Malignant melanoma; stage IV	51; M	20 weeks	Unknown	Unknown
Idogun et al., this article	NSCLC; stage IIb	67; F	19 weeks	Combined IVIG and intravenous methylprednisolone, followed by a weaning dose of oral prednisolone	Slow but complete recovery of neurological symptoms
Nivolumab and ipilimumab					
Supakornnumporn et al., 2017 [44]	Malignant melanoma; stage IV	77; M	10 weeks	IVIG 2 g/kg over five days, followed by prednisolone 90 mg/d	Significant improvement in neurological function
Pomerantz et al., 2019 [45]	SCLC; stage IV	58; M	8.5 weeks	Five days of IVIG 0.4 g/kg	Symptomatic improvement of neurological symptoms
McNeill et al., 2019 [46]	RCC; stage IV	68; M	8.5 weeks	Five-day course of IVIG (2 g/kg total); intravenous methylprednisolone (3 mg/kg/day) followed by oral prednisolone taper	Slow recovery of motor and sensory functions
Gu et al., 2017 [47]	Malignant melanoma; stage IV	49; F	5 days	IVIG, 0.4 g/kg/d for five days and intravenous methylprednisolone (1 g/d for five days, then 500 mg/d for three days) followed by tapering oral prednisolone (1 mg/kg/d).	Slow recovery of motor and sensory symptoms with relapsing
Baird-Gunning et al., 2018 [48]	Malignant melanoma; stage IV	58; F	10 days	Combined IVIG and intravenous methylprednisolone, followed by a weaning dose of oral prednisolone	Complete recovery in gait, ptosis, and extraocular movements. Remained areflexic

## Conclusions

Over the past several years, immunotherapy has changed the landscape of the available treatments for several oncologic malignancies. Despite having significantly less toxicity compared to traditional chemotherapy, ICIs are not without adverse side effects. NirAEs, including GBS, are an emerging area of interest because of the complexity of the nervous system and the potential for long-term morbidity. Early initiation of IVIG or plasma exchange has been proven to have benefits and is crucial, especially in patients with rapidly progressive weakness. Given the substantial risk of permanent neurological damage in the case of NirAEs, we do not recommend attempting retreatment with an ICI. There are not enough high-quality data or randomized controlled trials to draw absolute conclusions about the effects of PT in GBS, but in our case, it proved to be crucial in attaining complete neurologic recovery. Given the increasing use of ICI in cancer management, awareness of neurological autoimmune side effects cannot be overestimated due to associated fatalities if not diagnosed and managed properly.
